# Antiviral and Antioxidant Activity of a Hydroalcoholic Extract from *Humulus lupulus* L.

**DOI:** 10.1155/2018/5919237

**Published:** 2018-07-24

**Authors:** Antonella Di Sotto, Paola Checconi, Ignacio Celestino, Marcello Locatelli, Stefania Carissimi, Marta De Angelis, Valeria Rossi, Dolores Limongi, Chiara Toniolo, Lucia Martinoli, Silvia Di Giacomo, Anna Teresa Palamara, Lucia Nencioni

**Affiliations:** ^1^Department of Physiology and Pharmacology V. Erspamer, Sapienza University of Rome, P.le Aldo Moro 5, 00185 Rome, Italy; ^2^Department of Public Health and Infectious Diseases, Laboratory Affiliated to Istituto Pasteur Italia-Fondazione Cenci Bolognetti, Sapienza University of Rome, P.le Aldo Moro 5, 00185 Rome, Italy; ^3^IRCCS San Raffaele Pisana, Department of Human Sciences and Promotion of the Quality of Life, San Raffaele Roma Open University, Via di Val Cannuta 247, 00166 Rome, Italy; ^4^Department of Pharmacy, University “G. D'Annunzio” of Chieti-Pescara, Via dei Vestini 31, 66100 Chieti, Italy; ^5^Department of Biochemical Sciences “A. Rossi Fanelli”, Sapienza University of Rome, P.le Aldo Moro 5, 00185 Rome, Italy; ^6^Department of Environmental Biology, Sapienza University of Rome, P.le Aldo Moro 5, 00185 Rome, Italy

## Abstract

A hydroalcoholic extract from female inflorescences of *Humulus lupulus* L. (HOP extract) was evaluated for its anti-influenza activity. The ability of the extract to interfere with different phases of viral replication was assessed, as well as its effect on the intracellular redox state, being unbalanced versus the oxidative state in infected cells. The radical scavenging power, inhibition of lipoperoxidation, and ferric reducing activity were assayed as antioxidant mechanisms. A phytochemical characterization of the extract was also performed. We found that HOP extract significantly inhibited replication of various viral strains, at different time from infection. Viral replication was partly inhibited when virus was incubated with extract before infection, suggesting a direct effect on the virions. Since HOP extract was able to restore the reducing conditions of infected cells, by increasing glutathione content, its antiviral activity might be also due to an interference with redox-sensitive pathways required for viral replication. Accordingly, the extract exerted radical scavenging and reducing effects and inhibited lipoperoxidation and the tBOOH-induced cytotoxicity. At phytochemical analysis, different phenolics were identified, which altogether might contribute to HOP antiviral effect. In conclusion, our results highlighted anti-influenza and antioxidant properties of HOP extract, which encourage further *in vivo* studies to evaluate its possible application.

## 1. Introduction

Nowadays, influenza remains one of the main causes of morbidity worldwide, with seasonal epidemics and periodic pandemics. The disease is caused by an enveloped, RNA virus, which infects mainly the upper airways, but complications at lower respiratory tract can occur, especially in children and elderly [1].

Although different strategies have been approached to prevent the disease and/or manage its complications, the only anti-influenza drugs approved by FDA belong to two classes of inhibitors, which target the viral matrix protein 2 and neuraminidase [2]. Unfortunately, their efficacy is often limited by toxicity and by the emergence of novel drug-resistant viral mutants, thus requiring alternative and more effective therapeutic strategies [3]. Research in the field is looking for alternative molecules, both of natural and synthesis origin that could interfere with different targets, including cell host structures and pathways that virus exploits for its replication, and reduce the probability of drug resistance [4–6].

Several intracellular pathways that influenza virus activates are redox-sensitive [7, 8], and interestingly, some antioxidant molecules, including polyphenols, that are able to modulate the intracellular redox balance, also show anti-influenza activity [9–11]. Natural polyphenolic compounds, proanthocyanidins and catechins, have been found to possess antiviral properties, particularly against influenza A [12–14].

Female strobilus inflorescences (hops or cones) of *Humulus lupulus* L. (Fam. Cannabaceae) are widely used in the brewing industry as preservative and flavouring additives; in fact, *H. lupulus* is considered as an essential ingredient in beer, contributing to the bitter flavour and the characteristic hoppy aroma and providing preservative and antimicrobial effects [15–18]. Beer is often considered to be a “functional beverage” as a source of health-promoting substances [19]. Among them, polyphenolic compounds, mainly prenylflavonoids and procyanidins, along with chalcones (namely, xanthohumol) and the phytoestrogen 8-prenylnaringenin, have gained the most attention [17, 20]. Furthermore, *H. lupulus* extracts are used in traditional medicine as bitter stomachic and as remedies for mild sleeping disorders, in combination with other sedative herbs such as valerian, passion flower, and lemon balm [21].

*H. lupulus* and its polyphenolic constituents are reported to possess other interesting biological properties, including antimicrobial, antioxidant, anti-inflammatory, and chemopreventive activities [22–24]. Although antimicrobial (antibacterial and antifungal) properties are well known, little is reported about the antiviral activity of crude hop extracts and their purified components. Buckwold et al. [25] found that iso-*α*-acids and xanthohumol have a moderate antiviral activity against different RNA and DNA viruses, thus suggesting their possible role as lead compounds for more active antiviral agents.

In line with this evidence and our preliminary results [26], in the present study, the anti-influenza activity of a hydroalcoholic extract from the female inflorescences of *H. lupulus* (HOP extract) was evaluated. The ability of HOP to interfere with specific steps of viral replication was shown using an *in vitro* model, represented by permissive epithelial cell lines infected with different influenza A virus strains.

The HOP extract was characterized for its phenolic composition by both high-performance thin-layer chromatography (HPTLC) and high-performance liquid chromatography (HPLC-PDA), and the total polyphenol, tannin, and flavonoid amount was determined colorimetrically. Finally, the radical scavenger ability, the crocin bleaching activity, the inhibition of lipoperoxidation, and the iron reducing activity were evaluated as possible antioxidant and cytoprotective mechanisms of the HOP extract.

## 2. Materials and Methods

### 2.1. HOP Extract

A hydroalcoholic extract from the female inflorescences of *H. lupulus* L. (HOP extract; batch n. 1101385; code n. 3120004; ratio drug/extract 4 : 1), kindly supplied by EPO S.r.l. (Milan, Italy), was used to perform the experiments. The extract was standardized to contain 0.4% of flavonoids, determined as rutin equivalents.

### 2.2. Chemicals

All the chemicals, including 3-(4,5-dimethylthiazol-2-yl)-2,5-diphenyltetrazolium bromide (MTT; 98% purity), tert-butyl hydroperoxide (tBOOH, 70% wt in H_2_O), Triton X-100, 1,1-diphenyl-2-picryl-hydrazyl (DPPH; 95% purity), 2,2′-azino-bis(3-thylbenzothiazoline-6-sulfonic acid) diammonium salt (ABTS; 98% purity), 2,2′-azobis (2-methylpropionamidine) dihydrochloride (AAPH; 97% purity), ferrozine (97% purity), hydroxylamine hydrochloride (98% purity), iron(III) chloride (FeCl_3_ × 6H_2_O; 97% purity), iron(II) sulfate heptahydrate (FeSO_4_ × 7H_2_O; 99% purity), potassium hexacyanoferrate(III) (99.9% purity), iron(II) chloride (FeCl_2_ × 4H_2_O; 99% purity), Trolox (97% purity), standard phenolic compounds (>95% purity), the solvents ethanol (EtOH; 99.5% purity) and methanol (MeOH; 99.5% purity), and antiactin antibody were obtained from Sigma-Aldrich Co. (St. Louis, MO, USA). Sodium carbonate (Na_2_CO_3_; 99.999% purity), Folin-Ciocalteu's phenol reagent, tannic acid (Ph Eur purity), and aluminium chloride hexahydrate (AlCl_3_ × 6H_2_O; Ph Eur purity) were purchased from Merck (Darmstadt, Germany). Furthermore, the reagents for antiviral studies, if not otherwise specified, were purchased from Invitrogen (Carlsbad, CA, USA).

### 2.3. Phytochemical Analysis

#### 2.3.1. Chromatographic Analysis

HPTLC and HPLC-PDA phenolic pattern were evaluated according to previous standardized methods [27]. To perform the HPTLC analysis, the extract and the selected standard polyphenols rutin, chlorogenic acid, catechin, and gallic acid were dissolved in methanol at concentration of 30 mg/ml and 1 mg/ml, respectively. The phenolics were identified by comparison with the selected standards (Rf values, colors, and UV spectra).

For the HPLC-PDA, the HOP extract (20 *μ*l) was dissolved in the mobile phase (1 : 10 dilution factor) and injected into HPLC-PDA system. The standard phenolics, including benzoic acid, carvacrol, catechin, chlorogenic acid, epicatechin, gallic acid, harpagoside, naringenin, naringin, p-OH benzoic acid, quercetin, rutin, sinapinic acid, syringic acid, t-cinnamic acid, t-ferulic acid, and vanillic acid, were enclosed in the analysis.

#### 2.3.2. Total Polyphenols, Tannins, and Flavonoids

The total polyphenol, tannin, and flavonoid content was determined according to standardized spectrophotometric methods, with minor changes [27]. The total amount of both polyphenols and tannins was calculated as tannic acid equivalents (TAE), while flavonoids were expressed as quercetin equivalents (QE).

### 2.4. Antiviral Activity

#### 2.4.1. Cell Cultures

MDCK (Madin-Darby canine kidney) cells and A549 human lung carcinoma cells were grown in RPMI 1640 and DMEM medium, respectively, supplemented with 10% fetal bovine serum (FBS), 0.3 mg/ml glutamine, 100 U/ml penicillin, and 100 *μ*g/ml streptomycin.

#### 2.4.2. Cytotoxicity Assay

The cytotoxicity of the treatments was evaluated by using the 3-(4,5-dimethylthiazol-2-yl)-2,5-diphenyltetrazolium bromide reduction assay [28]. The HOP extract was dissolved in DMSO (concentrations range 20–180 *μ*g/ml) and added to both MDCK and A549 cells for 24 h. The cytotoxicity was calculated as percentage reduction in viability of HOP-treated cells compared to control, that is, cells treated with DMSO alone.

In the cytoprotection assay, after a 24 h pretreatment with the HOP extract, a low-toxic concentration (about 40% cytotoxicity as found in preliminary experiments) of the prooxidant agent tBOOH (5 *μ*M) was added for 2 h to cells, and then the cell viability was measured as described above.

#### 2.4.3. Viral Infection, Titration, and Viral mRNA Quantification

Confluent monolayers of MDCK or A549 cells were challenged with the following influenza A virus strains: human A/Puerto Rico/8/34 H1N1 (PR8), A/NWS/33 H1N1 (NWS), and pandemic A/California/04/09 H1N1 (pH1N1) strains or avian Parrot/Ulster/73 H7N1 (ULSTER) strain, at a multiplicity of infection (m.o.i.) of 3 (high m.o.i.) and 0.3 (low m.o.i.) for 1 h at 37°C. After the viral adsorption, the cells were washed with phosphate-buffered saline (PBS) and then incubated with medium supplemented with 2% FBS for 24 or 48 h.

For the evaluation of the antiviral activity, HOP extract was dissolved in DMSO and then diluted to the final concentrations in the cell culture medium. The highest DMSO concentration present in the culture medium was 0.2%. Control cells were treated with DMSO alone at the same concentration.

Treatment with HOP was performed as follows: 1 h before the infection (b.i.), during the 1 h adsorption period (d.i.), and after the adsorption (postinfection, p.i.) or before, during, and after the infection (b.d.p.i.).

To evaluate the virucidal effect, HOP extract was incubated directly with the virus for 1 h at 37°C. Then, the mixture was used to infect the cell culture as described above.

Virus production was determined in the supernatants of infected cells 24 and 48 h p.i., by measuring the hemagglutinating units (HAU) or the tissue culture infectious dose 50 (TCID50), as previously described [29].

For M1 mRNA quantification, total RNA was isolated from A549 cell lysates (Total RNA Purification Plus Kit, Norgen Biotek, Thorold, ON, Canada) and used as a template for generating cDNA (iScript cDNA Synthesis Kit, Bio-Rad, Milan, Italy). An aliquot of the cDNA was subjected to 40 cycles of RT-PCR amplification (95°C, 10 sec; 60°C, 30 sec) using iQ SYBR Green Supermix and a LightCycler iQ 5 (Bio-Rad, Milan, Italy). The housekeeping gene ribosomal protein L13A (Rpl13a) was used for normalization. Relative quantitative evaluation was performed by the comparative ΔΔCt method.

#### 2.4.4. Immunoblotting Analysis

Influenza virus-infected and HOP-treated (as described above) MDCK or A549 cells were lysed and analyzed by SDS-PAGE followed by Western blotting with anti-influenza (Merck Millipore, Darmstadt, Germany) and antiactin antibodies. HRP-linked anti-goat and anti-mouse (Jackson ImmunoResearch, Newmarket, UK) were used as secondary antibodies. The membranes were developed using Clarity Western ECL substrate (Bio-Rad, Hercules, CA, USA).

#### 2.4.5. Immunofluorescence Analysis

Following incubation of PR8 virus with HOP extract and infection with the mixture (or with PR8 alone), A549 cells were fixed with methanol, permeabilized with 0.1% Triton X-100 and stained with anti-NP antibody (Bio-Rad, Hercules, CA, USA). Alexa-Flour 488-conjugated anti-mouse was used as secondary antibody. Nuclei were stained with 4′,6-diamidino-2-phenylindole (DAPI).

#### 2.4.6. Glutathione Assay

GSH level was quantified in A549 cell lysates from PR8- or pH1N1-infected and HOP-treated (p.i. and b.d.p.i.) cells, as previously described [30]. The GSH levels were also measured in the lysates from cells treated with the prooxidant agent tBOOH (5 *μ*M). In these experiments, the antioxidant effect of the HOP extract was evaluated under pretreatment and cotreatment plus posttreatment protocols. Protein content was determined with Bradford reagent (Bio-Rad, Hercules, CA, USA) and GSH level expressed as nmol/mg proteins.

### 2.5. Antioxidant Activity

All tests were performed in 96-multiwell microplates away from direct light; the experiments were repeated at least twice, and in each experiment, each concentration was tested in triplicate. Data obtained from at least two experiments were pooled for statistical analysis.

To perform the assays, the HOP extract was dissolved in deionized water, and in each test, the suitable negative or positive controls (Trolox, rutin, and quercetin used as standard antioxidant agents) were included. The absorbance was measured by a microplate reader (Epoch Microplate Spectrophotometer, BioTek).

#### 2.5.1. Radical Scavenging Activity

DPPH and ABTS radical scavenging activities were determined according to Di Sotto et al. [27] with minor changes. Briefly, a DPPH solution (40 *μ*l; 0.1 mM in EtOH 100% *v*/*v*) and the test sample (160 *μ*l) were incubated for 30 minutes in the dark at room temperature, and then the absorbance of DPPH radical was measured at 517 nm. For the ABTS assay, equal volumes of ABTS (5 mM in PBS 0.1 M, pH 7.0) and AAPH (2 mM in PBS 0.1 M, pH 7.0) were mixed and incubated for 45 minutes at 68°C, to obtain the ABTS radical cation. The sample (20 *μ*l) was added to the radical solution (180 *μ*l), and the plates were incubated for 10 minutes in the dark at 37°C and then read at 734 nm. The percentage of scavenger activity was calculated as follows: 100 × (*A*_control_ − *A*_sample_)/*A*_control__,_ where *A*_control_ is the absorbance of the radical alone, while *A*_sample_ is that of radical with sample.

#### 2.5.2. Crocin Bleaching Assay

The assay was carried out according to Di Majo et al. [31] with minor changes. To perform the assay, a crocin solution (40 *μ*l; 3.5 mM in PBS 0.1 M, pH 7.4), AAPH (10 *μ*l; 0.25 M in PBS 0.1 M, pH 7.4), the extract (40 *μ*l), and PBS (110 *μ*l; 0.1 M, pH 7.4) were mixed; then the mixture was incubated at 40°C in the dark for 60 minutes. After incubation, the crocin absorbance was read at 443 nm. The percentage of antioxidant activity was calculated as follows: 100 × (*A*_control_ − *A*_sample_)/*A*_control__,_ where *A*_control_ is the absorbance of the crocin alone, while *A*_sample_ is that of radical with sample.

#### 2.5.3. Inhibition of Lipid Peroxidation

The assay was carried out by the ferric thiocyanate method according to Di Sotto et al. [32]. Briefly, the sample (125 *μ*l), PBS (500 *μ*l; 0.2 M, pH 7.0), and a linoleic emulsion (625 *μ*l) were incubated at 37°C for 96 h. Some aliquots (100 *μ*l) were taken every 24 h and further added with ethanol (470 *μ*l; 75% *v*/*v*), FeCl_2_ (10 *μ*l; 200 mM in 3.5% *w*/*v* HCl), and potassium hexacyanoferrate (KSCN, 10 *μ*l; 30% *w*/*v* in deionized water). Peroxides, released during linoleic acid peroxidation, oxidize ferrous to ferric ions, so forming a red ferric(III) thiocyanate complex, measured at 500 nm spectrophotometrically. The percentage of lipoperoxidation inhibition (LPI) was calculated as follows: [1–(*A*_sample_/*A*_control_)] × 100, where *A*_control_ was the absorbance of the vehicle while *A*_sample_ is that of the tested sample.

#### 2.5.4. Ferric Reducing Activity

The activity was evaluated by the ferrozine assay, according to previous published methods [27]. Briefly, equal volumes of FeCl_3_ × 6H_2_O (200 *μ*M in acetate buffer solution 0.1 M, pH 4.5) and the samples were mixed for 2 minutes, and then a ferrozine solution (5 mM in acetate buffer solution 0.1 M, pH 4.5) was added to the mixture. The ferrous ion-ferrozine complex, corresponding to the reducing activity of the sample, was measured at 562 nm and the percentage of activity was calculated as follows: 100 × (*A*_control_ − *A*_sample_)/*A*_control_, where *A*_control_ is the absorbance of the vehicle, while *A*_sample_ is that of the tested sample.

### 2.6. Statistical Analysis

For the antioxidant studies, all values are expressed as mean ± SE and *n* represents the number of experiences.

For the antiviral studies, all values are expressed as mean ± SD and *n* represents the number of replicates for each treatment.

Statistical analysis was performed by GraphPad Prism™ software (GraphPad Software Inc., San Diego, California, USA).

The one-way analysis of variance (one-way ANOVA), followed by Dunnett's multiple comparison posttest, was used to analyze the difference among different treatments, while the Student's *t*-test was applied to determine the statistical significance between two different experimental conditions. The values of *P* < 0.05 were considered significant.

The concentration-response curves were constructed using the “Hill equation”: *E* = *E*max/[1 + (10_50_^LogEC^/A)^HillSlope^], where *E* is the effect expressed as increase in the ferrous chelation at a given concentration of agonist, *E*max is the maximum ferrous chelating activity, IC_50_ is the concentration that produces a 50% of the inhibitory response, *A* is the agonist concentration in molar, and HillSlope is the slope of the agonist curve.

## 3. Results and Discussion

### 3.1. Phytochemical Analysis

The HPTLC analysis showed the presence in the chromatogram of different polyphenols, evidenced as fluorescent spots, and better visualized by derivatization with NPR and anisaldehyde (Figure S1). Among them, rutin, chlorogenic acid, and gallic acid were identified. The HPLC-PAD analysis confirmed the presence of rutin as one of the most abundant phenolic compounds, along with syringic acid and ferulic acid; lower levels were highlighted for p-OH benzoic acid, gallic acid, and chlorogenic acid (Table 1).

Colorimetric determinations highlighted a polyphenols/tannins ratio of about 4, while the flavonoid content agreed with that declared by the supplier (Table 2). On the basis of the DER, the total flavonoid content of the HOP extract resulted to be about 0.4% of the raw material. According to Peterson and Dwyer [33], which classified the flavonoid concentration in foods as low (0.1–39.9 mg/kg), moderate (40–99.9 mg/kg), and high (>100 mg/kg), the raw material resulted to contain a high flavonoid (i.e., 950 mg/kg) amount, thus suggesting its possible role as a nutraceutical source.

According to our results, Inui et al. [24] highlighted that syringic acid, along with procyanidins and catechins, was the major constituent of a hydroacetone extract from the *H. lupulus* plant, with lower levels of quercetin, naringenin, kaempferol, and xanthohumol. Ferulic acid was also found in a methanolic extract from cones of *H. lupulus* [34]. Furthermore, quercetin glycosides, such as rutin, isoquercitrin, and isoquercitrin malonate, have been identified as the main bioactive constituents of the water extract from hop plant [35]. Quercetin and kaempferol glycosides were also found in *H. lupulus* cones and young shoots [36, 37].

Our sample of HOP extract did not contain quercetin and naringenin, in spite of a high amount of rutin. This could be a consequence of the mild extraction condition (based on a maceration in ethanol 90% *v*/*v*), which allows a poor hydrolysis of the glycoside. In fact, the rutin hydrolysis has been found to occur under acidified conditions with high concentrated hydrochloric acid and extended heating times [38]. A significant influence of the extraction method on the phenolic concentration in *H. lupulus* extracts was reported: in spite of a very low phenolic amount in the aqueous extracts, the hydroalcoholic samples were found richer in epicatechin, chlorogenic acid, gallic acid, catechin, benzoic acid, ferulic acid, and o-coumaric acid [39]. The extraction method also affects the total amount of prenylflavonoids and bitter, being poorly stable under prolonged and hot extraction conditions [40].

### 3.2. Antiviral Activity

#### 3.2.1. HOP Extract Inhibits Influenza A Virus Replication in Different Phases of the Virus Life Cycle

**Figure 1:**
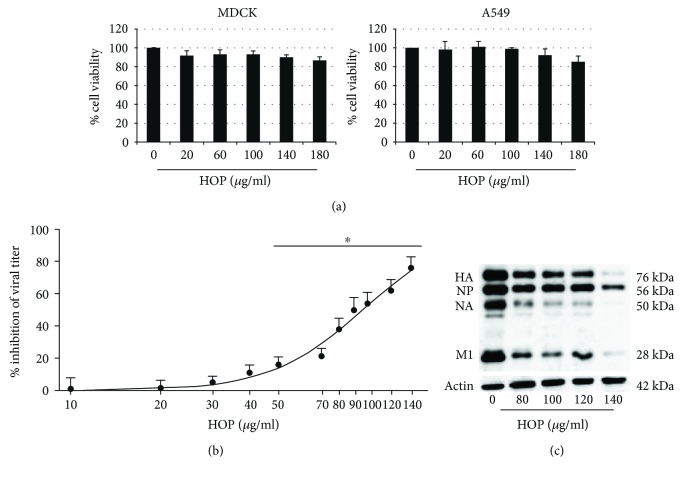
(a) Cell viability (percentage of control) of MDCK and A549 cells treated with different concentrations of HOP extract for 24 h as evaluated by MTT assay. (b) Percentage of inhibition of viral titer induced by HOP extract (10–140 *μ*g/ml) on MDCK cells infected with PR8 virus (low m.o.i.) and treated with HOP after infection for 24 h. Data are mean ± SD from three independent biological replicates, each one performed in two technical replicates (*n* = 3). ^∗^ *P* < 0.05 versus untreated infected cells by Student's *t*-test. (c) Western blot analysis of viral proteins (HA: hemagglutinin; NP: nucleoprotein; NA: neuraminidase; M1: matrix 1 protein) from PR8-infected MDCK cells, treated with HOP (80–140 *μ*g/ml) after infection for 24 h.

In order to study a possible antiviral activity of the HOP extract, first of all it has been necessary to rule out any cytotoxic effect in the tested cell lines. Using concentrations of the extract from 20 to 180 *μ*g/ml on MDCK and A549 epithelial cell lines, we demonstrated that the percentage of cellular viability was equal or higher than 90% compared to control cells till the concentration of 140 *μ*g/ml in both cell lines (Figure 1(a)). Since a slight reduction (about 13%) in cell proliferation was observed when cells were treated with HOP 180 *μ*g/ml, we chose to exclude this concentration for the following experiments.

Next, we tested the antiviral activity of different concentrations (10–140 *μ*g/ml) of HOP on kidney epithelial MDCK cells, well known to be highly permissive to influenza virus. As shown in Figure 1(b), the extract inhibited PR8 viral replication in a statistically significant (^∗^ *P* < 0.05 starting from 50 *μ*g/ml) and concentration-dependent manner, with an IC_50_ value of 99 (confidential limits 93–110) *μ*g/ml. Western blot analysis of viral proteins similarly showed a dose-dependent reduction of hemagglutinin (HA), neuraminidase (NA), nucleoprotein (NP), and matrix protein1 (M1) in infected and HOP-treated cells compared to the infected ones (Figure 1(c)).

**Figure 2:**
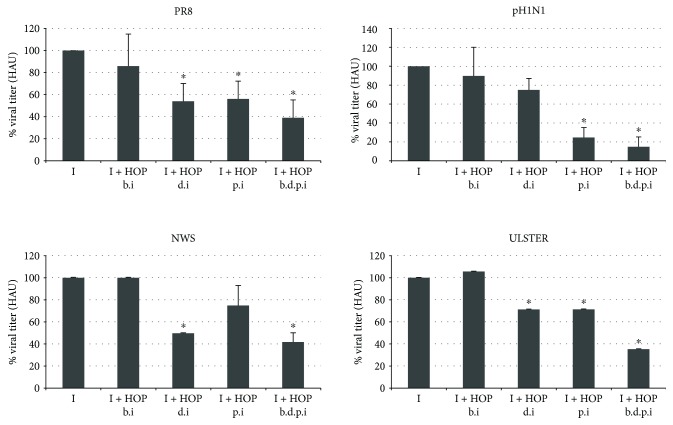
Viral titer measured in supernatants of A549 cells infected with different influenza A virus strains and treated with 140 *μ*g/ml HOP extract 1 h before the infection (b.i.), during the 1 h adsorption period (d.i.), and after the adsorption (postinfection: p.i.) or before, during, and after the infection (b.d.p.i.). Viral production was determined 24 h p.i. by hemagglutinating assay and expressed as percentage of HAU, (where titer from the untreated, infected cells was considered 100%). Data are mean ± SD from three independent biological replicates, each one performed in two technical replicates (*n* = 3). ^∗^ *P* < 0.05 versus untreated infected cells by Student's *t*-test.

On the basis of this preliminary evidence, the highest concentration of 140 *μ*g/ml was chosen for the antiviral assays in the human lung epithelial A549 cells. Particularly, these cells were infected with different strains of influenza A virus (PR8, pH1N1, NWS, and ULSTER) and treated with HOP at different times from the infection: before (b.i.), during (d.i.), and after (p.i.) or before, during, and after the infection (b.d.p.i). As shown in the charts of Figure 2, the treatment before the infection was ineffective. Instead, when the HOP extract was added to the culture medium with the virus during the 1 h infection, it was able to reduce viral titer, in a partial but significant degree for PR8, NWS, and ULSTER strains (46%, 50%, and 29% of inhibition, resp.). The HOP treatment performed after the infection significantly reduced pH1N1, PR8, and ULSTER titer (75%, 44%, and 29%, resp.). Interestingly, when the treatment was performed before, during, and after the infection, it significantly reduced viral titer of all the strains, suggesting an inhibitory additive effect of the HOP extract, when it was continuously present during all the time of infection.

#### 3.2.2. HOP Extract Exerts a Partial Virucidal Effect

**Figure 3:**
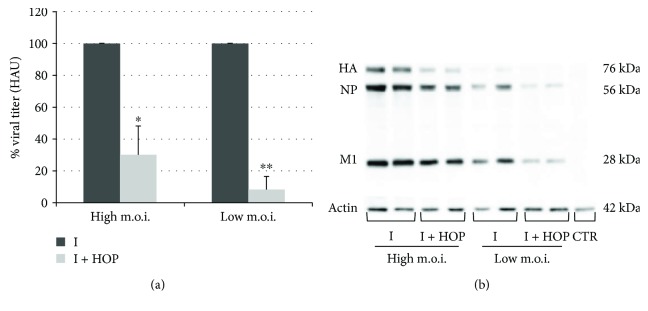
(a) Viral titer measured in supernatants of A549 cells infected with PR8 virus (high or low m.o.i.), following incubation of the virus with 140 *μ*g/ml HOP extract. Titer was determined 24 h p.i. by hemagglutinating assay and expressed as percentage of HAU compared to that from cells infected with untreated virus. Data are mean ± SD from three independent biological replicates, each one performed in two technical replicates (*n* = 3). ^∗^ *P* < 0.05 and ^∗∗^ *P* < 0.01 versus untreated infected cells by Student's *t*-test. (b) Western blot analysis of viral proteins from samples obtained as described in (a). Actin was used as loading control.

**Figure 4:**
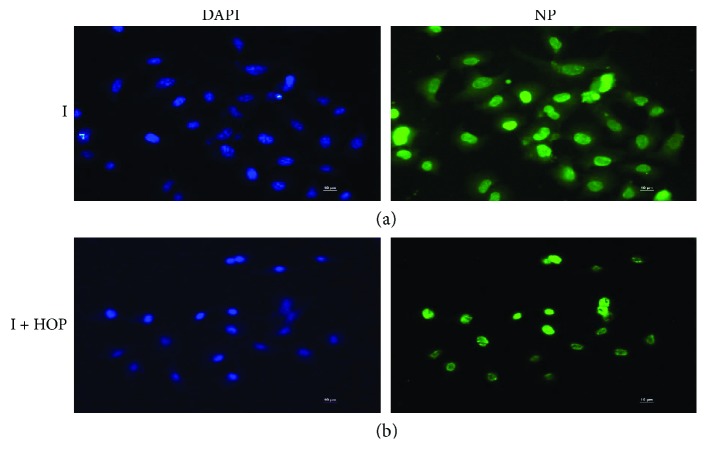
Immunofluorescence images of viral nucleoprotein (green fluorescence) in A549 cells infected for 24 h with PR8 virus (a) and following incubation of the virus with 140 *μ*g/ml HOP (b). Nuclei were stained with DAPI (blue).

In the attempt of explaining the molecular mechanism exerted by the HOP extract on influenza virus-infected cells, we decided to evaluate different steps of virus life cycle. First, we analyzed the effect of HOP extract during the 1 h infection. For this step, we could hypothesize a virucidal effect of the extract or an effect on an early phase of the viral life cycle. HOP extract was incubated directly with the virus and then the mixture used to infect the cells, as described in Materials and Methods. PR8 production, measured by the hemagglutinating assay, was significantly inhibited, both when high and low m.o.i. of virus were used, but with a higher percentage of inhibition when low m.o.i. was used (about 70% and almost 92% of inhibition, resp., Figure 3(a)). Results were confirmed by using the TCID50 assay, that is, the inhibition in viral replication was 80% when the extract was incubated with high m.o.i. of virus and over 90% when low m.o.i. of virus was used, confirming an inhibitory effect of the HOP extract that was viral dose-dependent, as it would be for a virucidal effect (Table 3). Western blot analysis of PR8 proteins obtained from lysates of cells infected with PR8-HOP mixture showed a lower expression of viral proteins compared to those in cells infected with the virus alone (Figure 3(b)). Similar results were obtained when cells were infected with other viral strains preincubated with HOP extract (see Figure S2). Furthermore, in these conditions, an immunofluorescence analysis of the viral nucleoprotein (NP) was performed using low m.o.i. of virus. According with Western blot results, the images in Figure 4 showed that NP was less expressed in cells infected with the virus-HOP mixture compared to the infected ones. Collectively, these results indicate that the HOP extract exerts at least a partial direct effect on viral particles, reducing their infectious ability. It has been already shown that the extract exerts an injurious effect on bacterial cell structures; in particular, its hydrophobic components are incorporated into the cell membranes, altering ion exchange [23]. Therefore, it is plausible that HOP components could be incorporated in the membrane of enveloped viruses, as influenza, destabilizing viral structure. However, we cannot exclude that HOP could enter the cells and act in an early phase of the cycle. Looking at the immunofluorescence images, the localization of NP appeared also affected, being in the nuclei of the HOP-treated infected cells, while it was diffused throughout the cell (nuclei plus cytoplasm) in untreated infected cells (Figure 4). This result suggests that the HOP components could also enter the cell and act by interfering with virus-activated cellular pathways, that in turn control NP traffic and localization.

**Figure 5:**
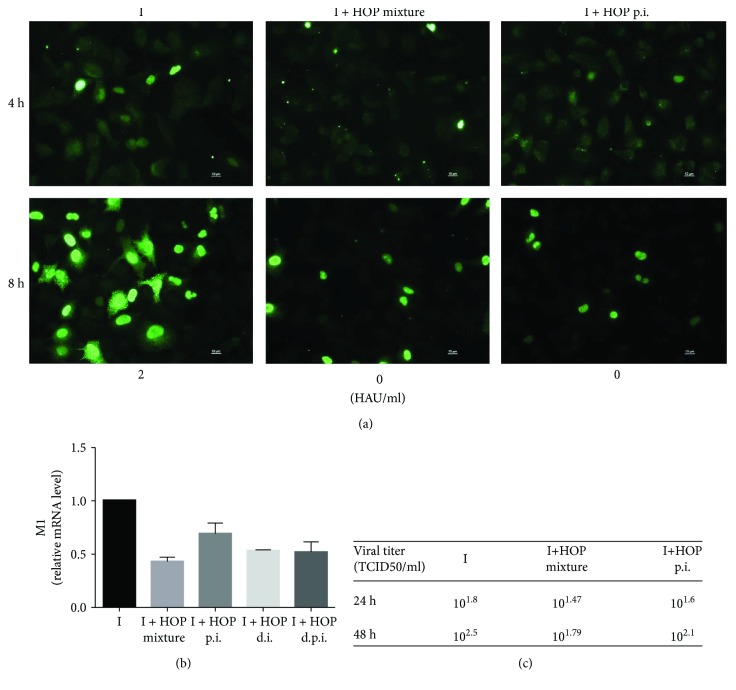
(a) Immunofluorescence images of viral nucleoprotein (green fluorescence) in A549 cells infected for 4 h and 8 h with PR8 virus (left panel), with PR8 and 140 *μ*g/ml HOP mixture (middle panel) or HOP treated after PR8 infection (right panel). HAU/ml values (8 h p.i.) are reported below the images. (b) M1 mRNA levels measured by qRT-PCR in A549 cells infected with PR8 virus for 2 h, with PR8/HOP mixture or HOP treated during (d.i.), after (p.i.), or during plus after (d.p.i.) infection. (c) TCID_50_ from supernatants harvested 8 h p.i. of same samples as in (a).

To deep inside the mechanisms hypothesized, we performed a second set of experiments during the first replication cycle of the virus. First of all, we repeated an immunofluorescence analysis of viral NP using high m.o.i. of virus for 4 h and 8 h of infection. As shown in Figure 5(a), left panel, NP was localized both in the nuclei and the cytoplasm of infected cells with an increased intensity from 4 h p.i. to 8 h p.i., accordingly with the progression of the viral life cycle. When the virus was preincubated with HOP (middle panel), the pictures appeared different, as a weak, diffused staining was visible with some brighter spot at 4 h p.i., which could represent viral particles sticking to the cell membranes and a lower number of infected cells at 8 h p.i. Moreover, in the cells that were infected, NP was localized mainly in the nuclei. When the cells were infected and treated with HOP after infection (right panel), the main effect was a different NP localization, being the viral protein mainly in the nuclei of infected cells at 8 h p.i.

qRT-PCR of mRNA levels of viral M1 performed at 2 h p.i. (Figure 5(b)) revealed a decrease in the expression of M1 mRNA in cells infected and HOP treated with different procedures, but especially with virus-HOP mixture, that could be the result of the reduced number of cells being infected. Accordingly, TCID50 from supernatants of infected cells 8 h p.i. showed higher inhibition when cells have been infected with virus-HOP mixture (Figure 5(c)).

#### 3.2.3. HOP Extract Restores the Intracellular GSH Levels of Influenza Virus-Infected Cells

**Figure 6:**
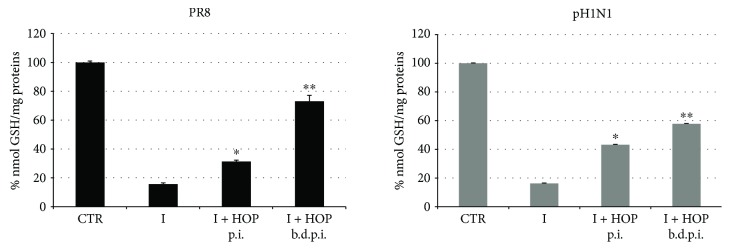
GSH levels measured in A549 cell lysates infected with PR8 or pH1N1 virus and treated with 140 *μ*g/ml HOP extract after the adsorption (postinfection: p.i.) or before, during, and after the infection (b.d.p.i.). Levels are expressed as percentage of nmol/mg proteins (where levels from control cells were considered 100%). Data are mean ± SD from three independent biological replicates (*n* = 3). ^∗^ *P* < 0.05 and ^∗∗^ *P* < 0.01 versus infected cells by Student's *t*-test.

In the present study, we also observed that HOP extract exerts an antiviral effect when added after the infection and especially before, during, and after the infection. These data let us to hypothesize that HOP could act also in another step of virus life cycle, with a further mechanism, that is, interfering with the redox imbalance caused by influenza virus. It is well known in fact that on the one hand, other *H. lupulus* extracts and some polyphenols exhibited antioxidant properties [23]; on the other hand, several viruses, including influenza, induce an oxidative stress to activate redox-sensitive pathways useful for their replication [8, 41] and a marker of redox changes is represented by glutathione (GSH) depletion in infected cells [29, 42]. Therefore, we measured the GSH levels in infected cells and HOP-treated after or before, during, and after the infection. Results were compared to infected and uninfected cells (control, CTR). As we expected, viral infection caused a significant GSH depletion (Figure 6). Interestingly, treatment with the HOP extract after the infection was able to restore GSH levels of about 30% and 40% (in PR8- and pH1N1-infected cells, compared to control). A more significant antioxidant effect was found when HOP extract was added before, during, and after the infection, and indeed, the GSH content reached 60% (in pH1N1-infected cells) and 73% (in PR8-infected ones) of that measured in control cells.

In the attempt to explain if the higher GSH content upon HOP treatment could be due to a reduced virus infection or a regulatory effect of the extract, the A549 cells were treated for 2 h with the prooxidant agent tBOOH after a 24 h pretreatment with the HOP extract. In these experimental conditions, tBOOH induced a slight but significant reduction of cell viability (about 20% lower than the vehicle) which disappeared in the presence of HOP (Figure S3a). These data were also confirmed in Caco-2 cells (Figure S3b), which represent a widely standardized model for studying oxidative stress-induced toxicity and the effect of dietary antioxidants [43]. Also, the GSH levels were markedly reduced by tBOOH (at least 70% reduction respect to the vehicle in all the experimental conditions), as confirmed by the cell morphology changes (Figures S4 and S5). Adding the HOP extract to cells, both under pretreatment and cotreatment plus posttreatment protocols, was able to partly counteract the tBOOH-induced oxidative toxicity and the cell morphology change (Figures S4 and S5). This evidence confirms our hypothesis about the antioxidant power of the tested sample, although the higher GSH levels found in the PR8-infected cells with respect to those damaged by tBOOH suggest that combined virucidal and antioxidant mechanisms can contribute to the antiviral properties of the HOP extract. Overall, the results indicate that HOP extract actually acts as antioxidant into infected cells. It could buffer reactive oxygen species (ROS), which are produced during infection [44], and in this way, it could reduce GSH depletion. This event in turn could block some redox-regulated cell pathways important for influenza virus replication. A lot of polyphenols have been shown able to inhibit for example phosphorylation of MAP kinases, including p38 MAPK and ERK, that are involved in the NP traffic [4, 9, 45]. Further studies are needed to distinguish the molecular pathways involved in the HOP extract antiviral effect.

### 3.3. Antioxidant Activity

During influenza virus infection, the NADPH oxidase isoform 4 (NOX4) is the main source of ROS production [8]. Indeed, inhibition of NOX4 activity or RNA silencing for this enzyme, by blocking ROS increase, prevents MAPK phosphorylation and inhibits NP traffic and viral release. In order to clarify the possible mechanisms by which the HOP extract exerted antioxidant effects in the influenza virus-infected cells, different *in vitro* antioxidant assays were performed. The radical scavenging properties of the sample, based on hydrogen and electron transfer, were evaluated against the synthetic chromogenic DPPH and ABTS radicals. Particularly, the HOP extract (0.001–2 mg/ml) inhibited, in a significant and concentration-dependent manner, both DPPH and ABTS radicals, as confirmed by the IC_50_ values (Table 4). The positive control Trolox was about 115- and 150-fold more effective against DPPH and ABTS, respectively. According to the Pearson analysis, these activities were significantly correlated (Table 5), although the sample was most potent (almost twofold) against ABTS, likely suggesting a major involvement of the electronic transfer in the scavenger activity. Taking into account that DPPH and ABTS radicals strongly differ for chemical structures, affinities, and kinetics of reaction, a higher affinity of the HOP constituents for ABTS can be hypothesized. A hydroalcoholic extract from *H. lupulus* cones exhibited radical scavenging effects of DPPH radical at higher concentrations with respect to hexane and methanol extract, although it contained high levels of gallic acid, quercetin, and kaempferol derivatives: this suggests that these compounds could not be the main responsible for the radical scavenging properties of the extract [34]. A weakly DPPH scavenging activity was also reported for the leaves of *Humulus lupulus* [46].

**Figure 7:**
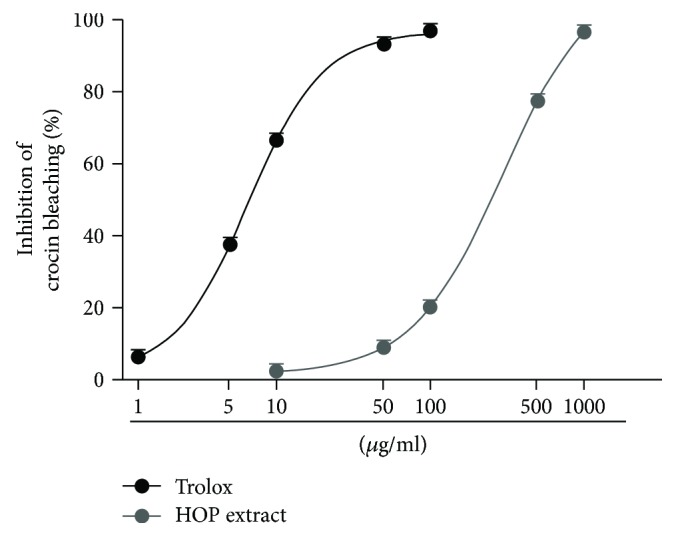
Effect of HOP extract on the AAPH-induced crocin bleaching after 24 h of incubation. Data are mean ± SE from two independent experiments, each one performed in two technical replicates (*n* = 4).

Under our experimental condition, the HOP extract also exhibited antioxidant effect in the crocin bleaching assay (Figure 7), being the IC_50_ value about 44-fold higher than that of Trolox (Table 4). Crocin bleaching represents a common antioxidant method, which uses crocin as the substrate and AAPH as a source of free radicals: the antioxidant competes with crocin and interferes with the bleaching of crocin. This assay is classified among those that involve the transfer of one hydrogen and is suitable for aqueous systems [47]. On the basis of the Pearson analysis, the crocin bleaching inhibition by extract resulted significantly correlated with the scavenger activity of both DPPH and ABTS radicals (Table 5). Although both DPPH and crocin bleaching assays are based on the hydrogen transfer for blocking radicals, the HOP extract resulted most potent against the crocin-derived radicals respect to DPPH radical. This difference could be due to the reaction media required for each assay (an aqueous medium for crocin solubilization, while methanol or ethanol for DPPH) [48], in which certain bioactive compounds can be low soluble and thus weakly effective. In this context, the aqueous medium seems to favor the radical scavenging properties of the HOP extracts, thus suggesting the presence of hydrosoluble bioactive constituents.

**Figure 8:**
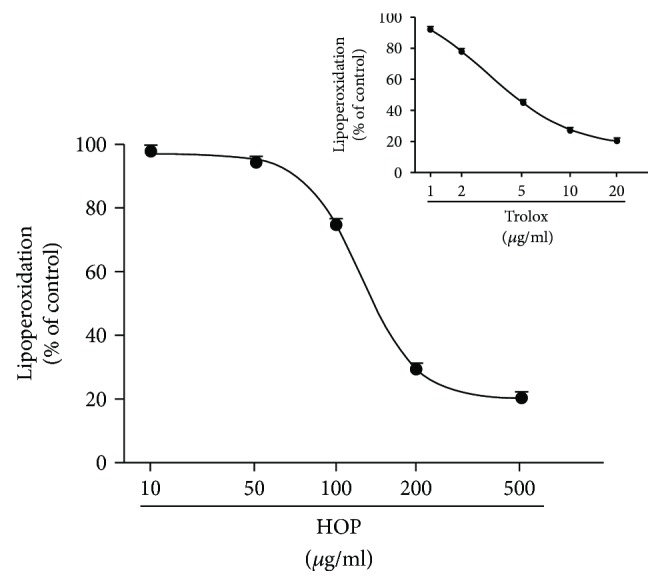
Effect of HOP extract on the linoleic acid oxidation (24 h of incubation) as measured by the ferric thiocyanate method. Data are mean ± SE from two independent experiments, each one performed in two technical replicates (*n* = 4).

The HOP extract was also found able to inhibit the lipid peroxidation in the ferric thiocyanate assay, already at low concentrations (Figure 8), as confirmed by the IC_50_ value (Table 4). Lipid peroxidation consists of a series of free radical-mediated chain reaction processes and is associated with several types of biological damage [48]. The ferric thiocyanate method, here used, measures the amount of peroxides, which are produced by lipid oxidation during the initial stages of ROS damage. In this assay, hydroperoxides are produced from the autoxidization of linoleic acid and are measured indirectly by the formation of ferric thiocyanate complex. According to the Pearson analysis, the inhibition of lipoperoxidation by HOP extract resulted significantly correlated with its radical scavenger activity (Table 5), thus suggesting that blocking the radical species can prevent the induction of lipid peroxidation. Taking into account that lipid peroxidation represents a major form of cellular oxidation damage, initiated by hydroxyl free radical through the extraction of hydrogen atom from unsaturated fatty acids of membrane phospholipids [49], we can hypothesize that HOP extract can interfere with the peroxidation of the cell biomembranes (by direct ROS neutralization or by blocking their generation) and prevent their structural changes, thus resulting in cytoprotective effects.

Under our experimental conditions, the extract resulted also able to reduce the ferric ions, although with a lower potency (about 140-fold) than the positive control Trolox (Table 4). As estimated by the Pearson analysis, the Fe^3+^ reducing activity appeared to be related with the lipoperoxidation inhibition (Table 5). ROS species production can be also facilitated by elemental species, such as iron, involved in metal-catalysed oxidation of biological substrates and in oxygen reactive species generation [48]. Ferric reducing power, based on the electron donating capacity of the antioxidant, represents a further antioxidant and cytoprotective mechanism, since reducing species can be oxidized in place of biological substrates [50]. In this context, our results showed that the HOP extract contains reducing species, which can be involved in the inhibition of lipid peroxidation. This effect should also contribute to counteract the redox imbalance induced by viral infections, thus mediating both cytoprotective and antiviral effects. Accordingly, the Pearson analysis indicated that the antiviral activity of HOP extract was significantly correlated with its antioxidant properties, particularly Fe^3+^ reducing activity and lipoperoxidation inhibition (Table 6).

Taken together, present results suggest that the HOP extract is able to interfere both directly and indirectly with the ROS-mediated cell injury. It is also plausible that HOP extract might interfere with NOX4 enzyme or with downstream pathways that are activated by oxidative stress.

Antioxidant properties of *H. lupulus* have been widely ascribed to its phenolic constituents. The most significant antioxidant activities are displayed by prenylated chalcones (i.e., xanthohumol) mainly due to their prenyl group [51]. Prenylflavonoids were reported able to chelate bivalent metals, thereby inhibiting the ROS generation. On the other hand, the antioxidant properties of different polyphenols, such as quercetin, chlorogenic acid, syringic acid, benzoic acid, catechin, and epigallocatechin, have been published [24, 52]. Therefore, all these compounds can contribute to the biological activity of the extract.

## 4. Conclusion

In the present study, we described the antiviral properties of a hydroalcoholic extract from the female inflorescences of *H. lupulus*, focusing on its ability to both directly counteract the viral replication and viral protein synthesis and indirectly increase the hosting cell defense by antioxidant mechanisms, likely due to its phenolic content.

Very limited evidence on the antiviral properties of *H. lupulus* and its characteristic constituents is available in literature. Buckwold et al. [25] found no antiviral effects of hop crude extracts and its chalcones against the influenza A and B viral strains.

*H. lupulus* cones represent an important source of phenolic compounds, some of which are reported to produce inhibitory effects against several viral infections [53–58]. Particularly, among the polyphenols identified in the HOP extract, rutin and quercetin were reported to inhibit the influenza infection in animal models, as well as the viral neuraminidase activities in vitro [59–61] and syringic acid and gallic acid were found to be potent anti-influenza compounds [62, 63]. This evidence supports our hypothesis about the possible involvement of the phenolic constituents in the antiviral properties of the HOP extract. Particularly, rutin, syringic acid, and gallic acid appear to be the potential bioactive constituents, although the contribution of all the phytocomplex cannot be excluded. Altogether, these compounds can act by both interfering with the virion life cycle and by reinforcing the defenses of hosting cells, mainly counteracting the redox imbalance required for the viral infection establishment.

In conclusion, our results suggest that the HOP extract is able to exert dual antiviral and cytoprotective effects, which can be useful in the prevention and treatment of influenza. Further investigations are encouraged in order to define the possible application of the HOP extract as an anti-influenza remedy or in combination with conventional antiviral drugs.

## Figures and Tables

**Table 1:** Phenolic composition of the HOP extract by HPLC-PDA analysis.

Compounds	Concentration (*μ*g/mg of the sample) (mean ± SD)
Benzoic acid	nd
Carvacrol	nd
Catechin	nd
Chlorogenic acid	0.017 ± 0.001
t-Cinnamic acid	nd
Epicatechin	nd
t-Ferulic acid	0.078 ± 0.005
Gallic acid	0.026 ± 0.002
Harpagoside	nd
Naringenin	nd
Naringin	nd
p-OH benzoic acid	0.029 ± 0.002
Quercetin	nd
Rutin	0.363 ± 0.001
Sinapinic acid	nd
Syringic acid	0.289 ± 0.020
Vanillic acid	BLD

BLD: below limit of detection; nd: not detected.

**Table 2:** Amounts of total polyphenols, tannins, and flavonoids in the HOP extract (*n* = 3).

Compound	HOP extract (*μ*g/mg of the sample) (mean ± SE)
Total polyphenols (TA equivalents)	7.1 ± 0.35
Tannins (TA equivalents)	1.7 ± 0.06
Flavonoids (Q equivalents)	3.8 ± 0.59

TA: tannic acid; Q: quercetin.

**Table 3:** TCID_50_ from supernatants of A549 cells infected with PR8 virus following incubation of the virus with 140 *μ*g/ml HOP.

Viral titer TCID50/ml	I high m.o.i.	I + HOP high m.o.i.
24 h	1585	316
48 h	3162	631

	I low m.o.i.	I + HOP low m.o.i.
24 h	ND	ND
48 h	35481	3162

**Table 4:** IC_50_ values of the HOP extract and the standard antioxidant agent Trolox in the antioxidant assays.

	HOP extract	Trolox
	IC_50_ (CL) *μ*g/ml	
DPPH scavenging activity	574.1 (392.1–840.3)	5.0 (4.4–5.8)
ABTS scavenging activity	311.1 (724.5–1146.2)	2.1 (1.6–2.4)
Inhibition of crocin bleaching	288.6 (270.8–320.4)	6.6 (3.7–11.5)
Lipoperoxidation inhibition	124.1 (91.2–168.7)	3.0 (1.1–7.6)
Fe^3+^ reducing activity	210.9 (110.5–329.3)	1.5 (1.1–2.0)

**Table 5:** Pearson correlation coefficient among antioxidant activity assays for the HOP extract.

	Pearson's *r* (CL; *R* ^2^)				
	DPPH scavenger activity	ABTS scavenger activity	Crocin bleaching inhibition	Lipoperoxidation inhibition	Fe^3+^ reducing activity
DPPH scavenger activity	1	—	—	—	—
ABTS scavenger activity	0.97^∗∗^ (0.83–0.99; 0.95)	1	—	—	—
Crocin bleaching inhibition	0.96^∗∗^ (0.53–0.99; 0.93)	0.99^∗∗^ (0.86–0.99; 0.98)	1	—	—
Lipoperoxidation inhibition	0.96^∗^ (0.23–0.99; 0.92)	0.99^∗∗^ (0.81–0.99; 0.99)	0.99^∗∗^ (0.3–0.99; 0.99)	1	—
Fe^3+^ reducing activity	nsc	nsc	nsc	0.88^∗^ (0.3–0.92; 0.78)	1

^∗^ *P* < 0.05 or ^∗∗^ *P* < 0.01, statistically significant correlation (two-tailed *t*-test). nsc: not significantly correlated; CL: confidential limits.

**Table 6:** Pearson correlation coefficient between antiviral activity and antioxidant activity of the HOP extract.

Antiviral activity	Pearson's *r* (CL; *R* ^2^)
DPPH scavenger activity	nsc
ABTS scavenger activity	nsc
Crocin bleaching inhibition	0.99^∗∗^ (0.53–0.99; 0.98)
Inhibition of lipoperodixation	0.99^∗∗^ (0.63–0.99; 0.98)
Fe^2+^ chelating activity	0.99^∗∗^ (0.88–0.99; 0.99)

^∗∗^ *P* < 0.01, statistically significant correlation (two-tailed *t*-test). nsc: not significantly correlated; CL: confidential limits.

## Data Availability

The high-performance thin-layer chromatography (HPTLC) of the HOP extract and its effect on replication of different influenza viral strains, on the oxidative damage and GSH level induced by tert-butyl hydroperoxide in A549 cells are available in the Supporting Materials.

## Supplementary Materials

Supplementary Materials Figure S1: high-performance thin-layer chromatography (HPTLC) of the polyphenolic compounds of the HOP extract. (a) Visualization at 366 nm without derivatization. (b) Visualization at 366 nm after natural product reagent (NPR) derivatization. (c) Visualization under white light after NPR and anisaldehyde derivatization. (d) Visualization at 366 nm after anisaldehyde/NPR derivatization. (e) Visualization at 255 nm. Figure S2: effect of the HOP extract on replication of different influenza viral strains in A549 cells. (a) Viral titer measured in supernatants of A549 cells infected with pH1N1, NWS, or ULSTER strain, following incubation of the virus with 140 *μ*g/ml of HOP extract. (b) Western blot analysis of viral proteins from samples obtained as described in (a). Figure S3: effect of the HOP extract (60–140 *μ*g/ml) on the oxidative damage induced by tert-butyl hydroperoxide (tBOOH; 5 *μ*M) in A549 (a) and Caco-2 cells (b). Figure S4: GSH levels measured in A549 cell lysates treated with tBOOH or tBOOH and HOP extract (140 *μ*g/ml) under pretreatment (a) and cotreatment plus posttreatment (b) protocols. Figure S5: morphology of A549 cells treated with tBOOH or tBOOH and HOP extract (140 *μ*g/ml) under the cotreatment plus posttreatment protocol.
