# Supplementary material for: Antiviral and Antioxidant Activity of a Hydroalcoholic Extract from Humulus lupulus L
Source: Oxid Med Cell Longev. 2018 Jul 24;2018:5919237. doi: 10.1155/2018/5919237 (PMC6081516; doi:10.1155/2018/5919237)

Supplementary materials

Antiviral and antioxidant activity of a hydroalcoholic extract from *Humulus lupulus* L.

Antonella Di Sotto[a,*], Paola Checconi[b,c,*], Ignacio Celestino[b,c], Marcello Locatelli[d], Stefania Carissimi[e], Marta De Angelis[b], Valeria Rossi[b], Dolores Limongi[c], Chiara Toniolo[f], Lucia Martinoli[a], Silvia Di Giacomo[a], Anna Teresa Palamara[b,c], Lucia Nencioni[b]

[a] Department of Physiology and Pharmacology, Sapienza University, P.le Aldo Moro 5, 00185, Rome, Italy

[b] Department of Public Health and Infectious Diseases, Sapienza University of Rome, Laboratory affiliated to Istituto Pasteur Italia-Fondazione Cenci Bolognetti, P.le Aldo Moro 5, 00185, Rome, Italy

[c] IRCCS San Raffaele Pisana, Department of Human Sciences and Promotion of the Quality of Life, San Raffaele Roma Open University, Via di Val Cannuta 247, 00166, Rome, Italy

[d] Department of Pharmacy, University "G. D'Annunzio" of Chieti-Pescara, Via dei Vestini 31, 66100, Chieti, Italy

[e] Department of Biochemical Science "A. Rossi Fanelli", Sapienza University of Rome, P.le Aldo Moro 5, 00185 Rome, Italy

[f] Department of Environmental Biology, Sapienza University, P.le Aldo Moro 5, 00185 Rome, Italy

*Corresponding authors:

Antonella Di Sotto: antonella.disotto@uniroma1.it

Paola Checconi: paola.checconi@uniroma1.it

Antonella Di Sotto and Paola Checconi equally contributed to this work.

Figure S1: High-performance thin-layer chromatography (HPTLC) of the polyphenolic compounds of the HOP extract. (a) Visualization at 366 nm without derivatization. (b) Visualization at 366 nm after Natural Product Reagent (NPR) derivatization. (c) Visualization under white light after NPR and anisaldehyde derivatization. (d) Visualization at 366 nm after anisaldehyde/NPR derivatization. (e) Visualization at 255 nm. The chromatograms correspond to: HOP extract (30 mg/mL; 1 μl; tracks 1,2), standard polyphenols (1 mM; 1 μL) rutin (track 3), chlorogenic acid (track 4), catechin (track 5), gallic acid (track 6).

a

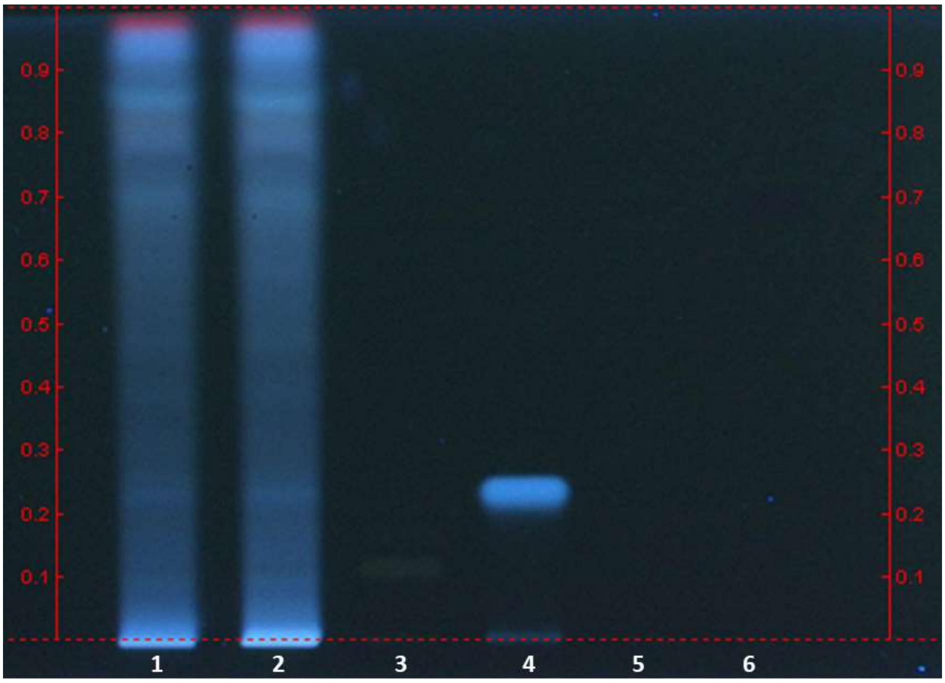

b

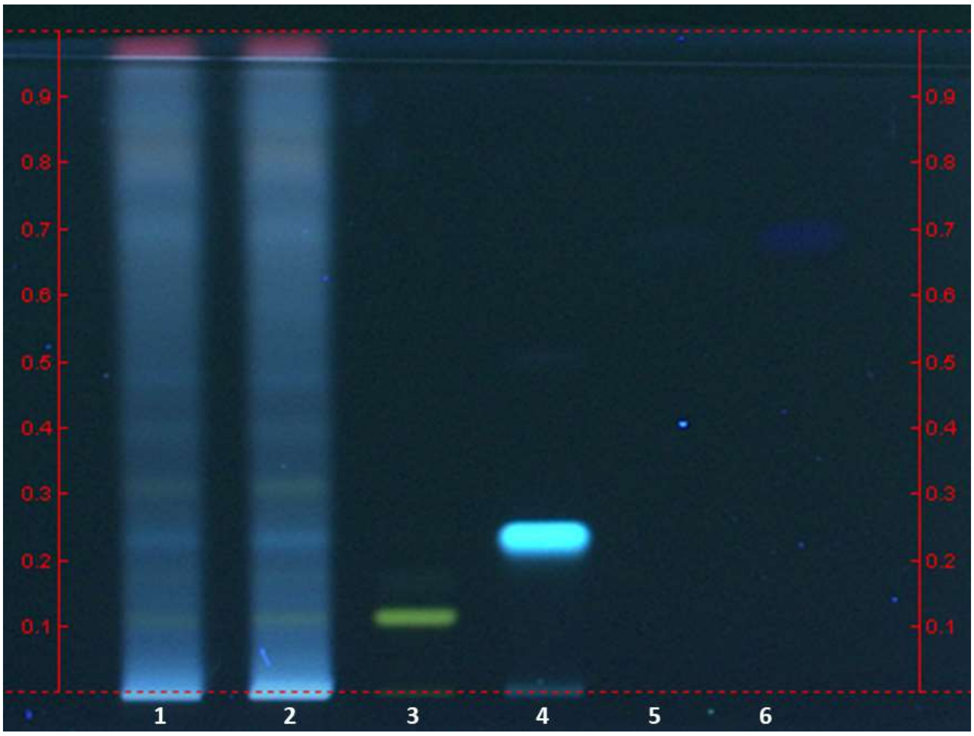

c

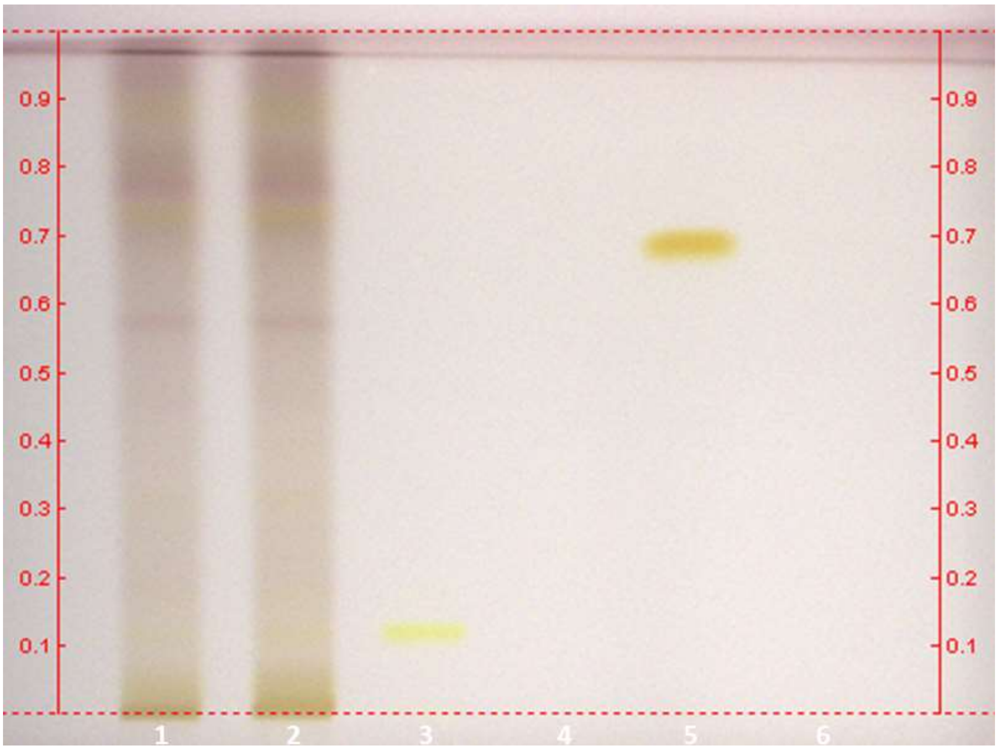

d

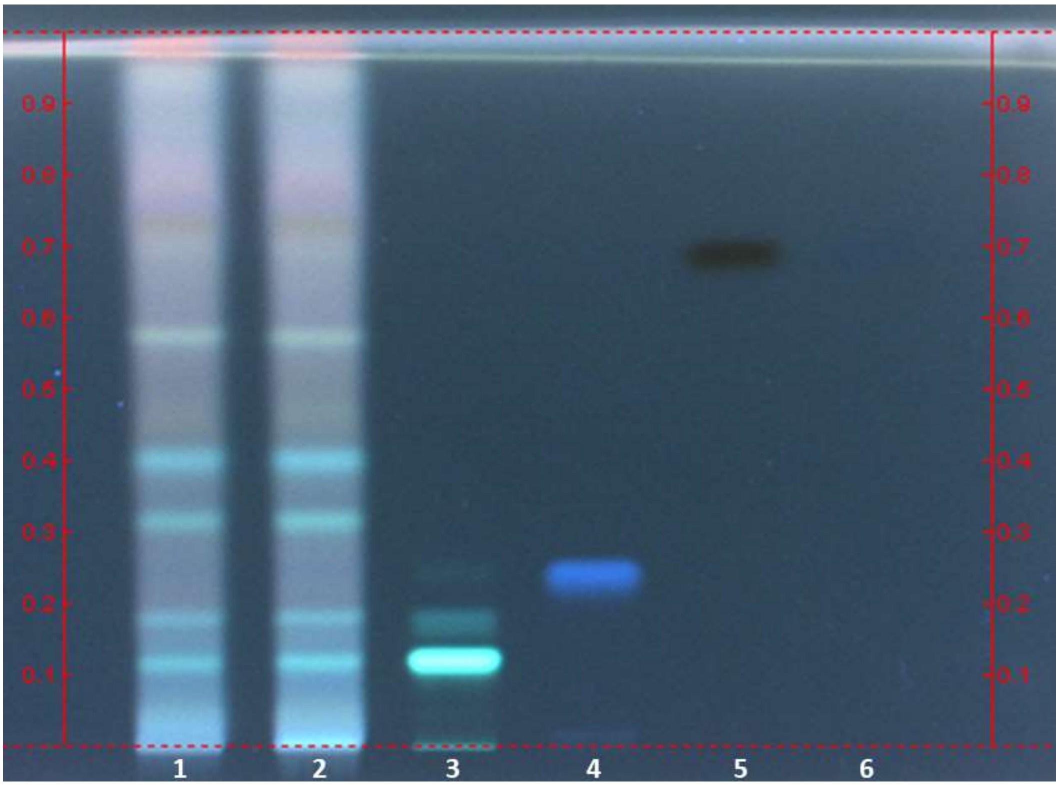

e

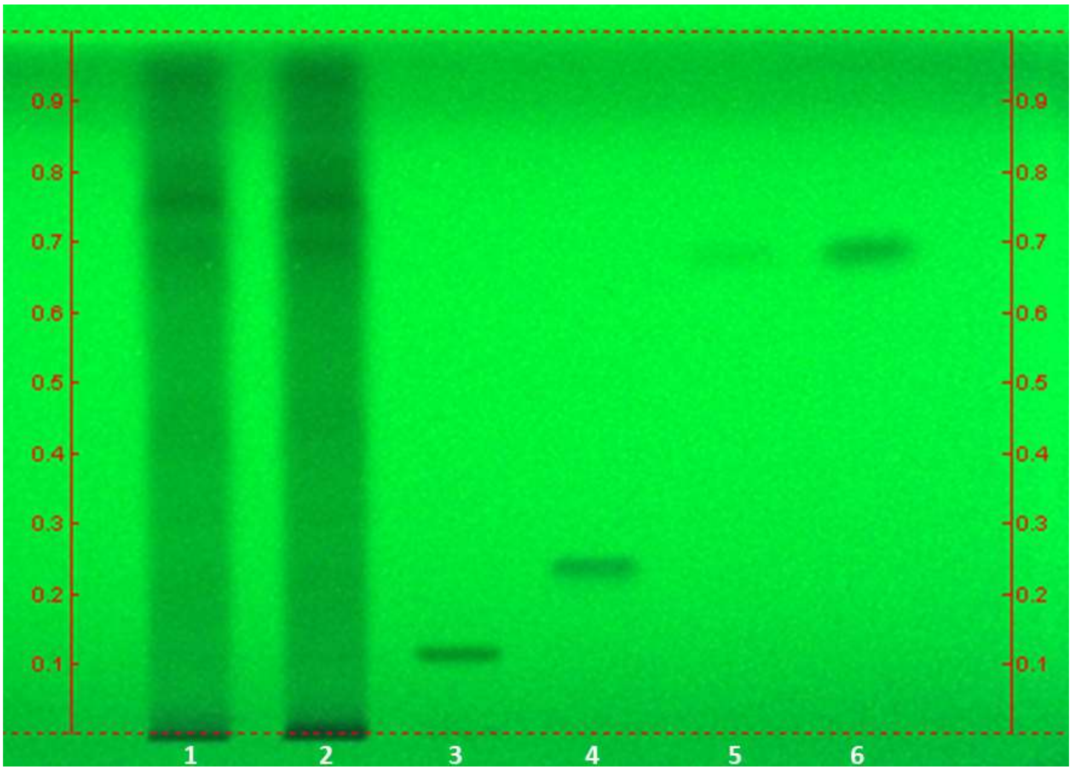

Figure S2: Effect of the HOP extract on replication of different influenza viral strains in A549 cells. (a) Viral titer measured in supernatants of A549 cells infected with pH1N1, NWS or ULSTER strain, following incubation of the virus with 140 μg/mL of HOP extract. Titer was determined 24 hrs p.i. by hemagglutinating assay and expressed as percentage of HAU compared to that from cells infected with untreated virus. Data are mean ± SD from two independent biological replicates, each one performed in two technical replicates (n = 2). * $P < 0.05$ *vs* untreated infected cells by Student's t-test. (b) Western blot analysis of viral proteins from samples obtained as described in (a). Actin was used as loading control.

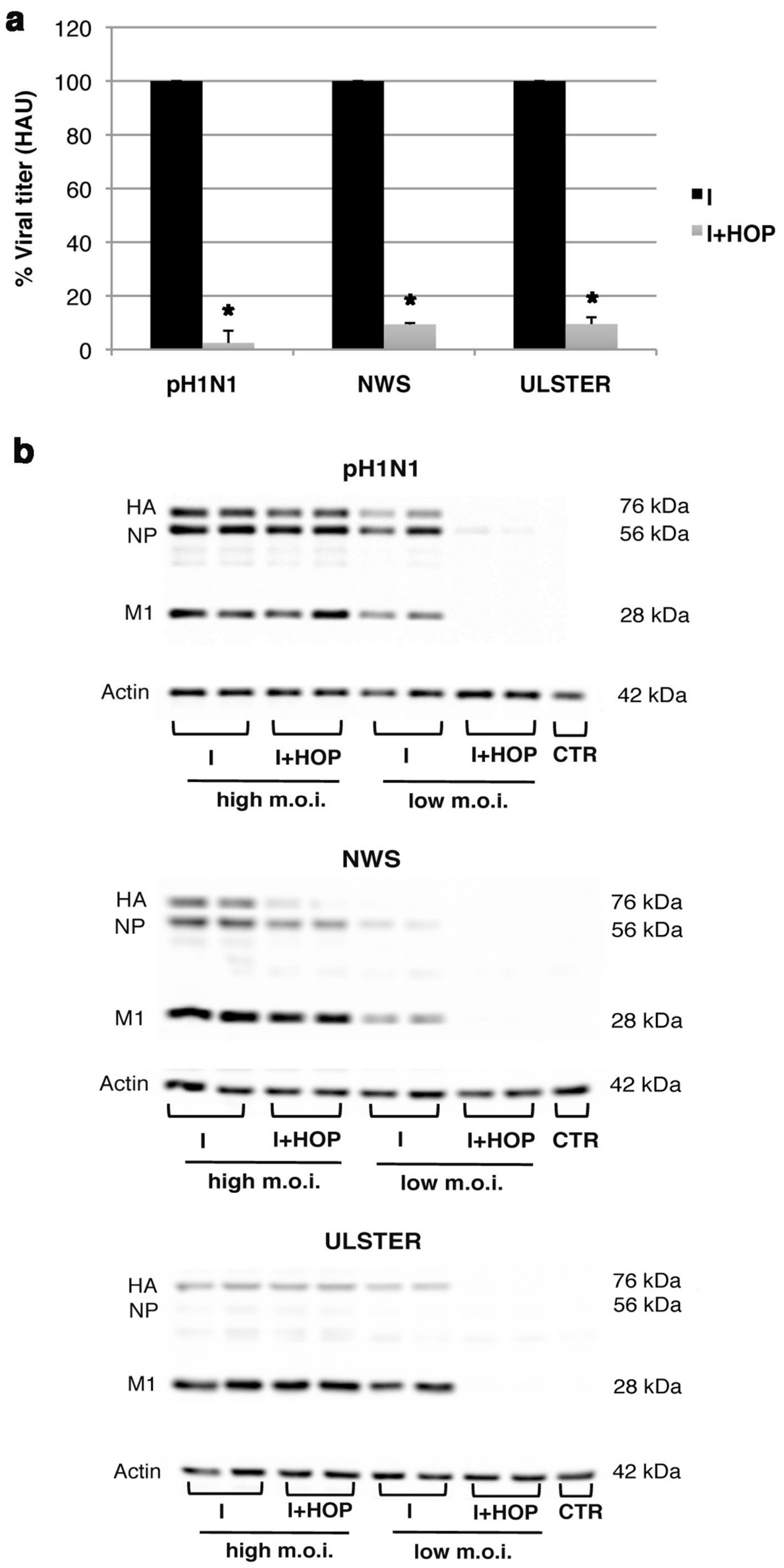

a
120
100
80
60
40
20
0
% Viral titer (HAU)
pH1N1
NWS
ULSTER
I
I+HOP
b
pH1N1
HA
NP
M1
Actin
76 kDa
56 kDa
28 kDa
42 kDa
I
I+HOP
I
I+HOP
CTR
high m.o.i.
low m.o.i.
NWS
HA
NP
M1
Actin
76 kDa
56 kDa
28 kDa
42 kDa
I
I+HOP
I
I+HOP
CTR
high m.o.i.
low m.o.i.
ULSTER
HA
NP
M1
Actin
76 kDa
56 kDa
28 kDa
42 kDa
I
I+HOP
I
I+HOP
CTR
high m.o.i.
low m.o.i.

Figure S3: Effect of the HOP extract (60 - 140 μg/mL) on the oxidative-damage induced by tert butyl hydroperoxide (tBOOH; 5 μM) in A549 (a) and Caco2 cells (b). The pro-oxidant agent tBOOH was added to cells after a 24 hrs pre-treatment with the HOP extract. Cell viability expressed as % of control. Data are mean ± SD from two independent biological replicates, each one performed in two technical replicates (n = 2). ** $P < 0.01$ vs tBOOH by ANOVA followed by Dunnett's Multiple Comparison Post Test.

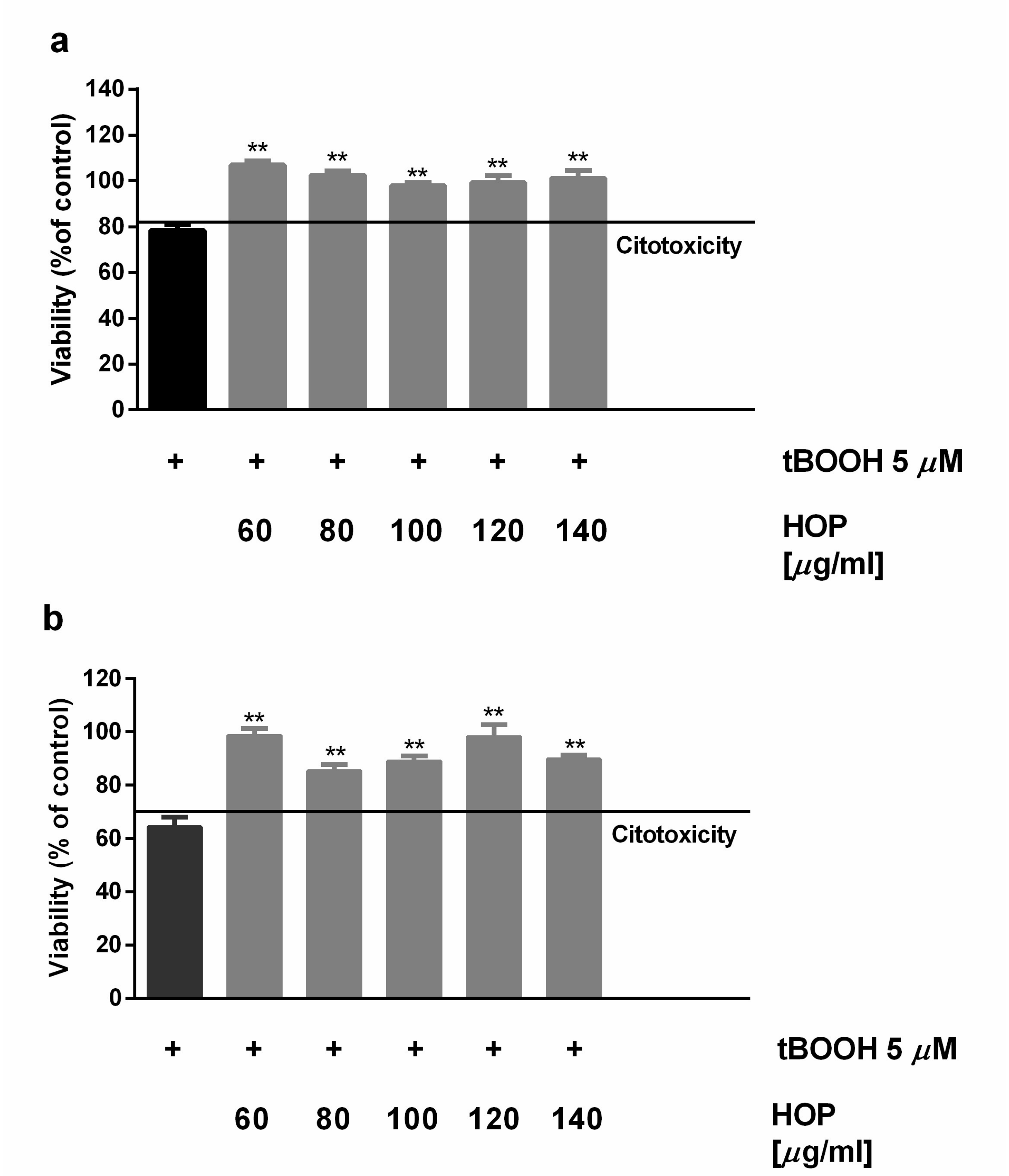

Figure S4: GSH levels measured in A549 cell lysates treated with tBOOH or tBOOH and HOP extract (140 μg/mL) under pre-treatment (a) and co-treatment plus post-treatment (b) protocols. Levels are expressed as percentage of nmoles/mg proteins (where levels from control cells were considered 100%). Data are mean ± SD from almost two independent biological replicates (n = 2). ** $P < 0.01$ vs tBOOH by Student's t-test.

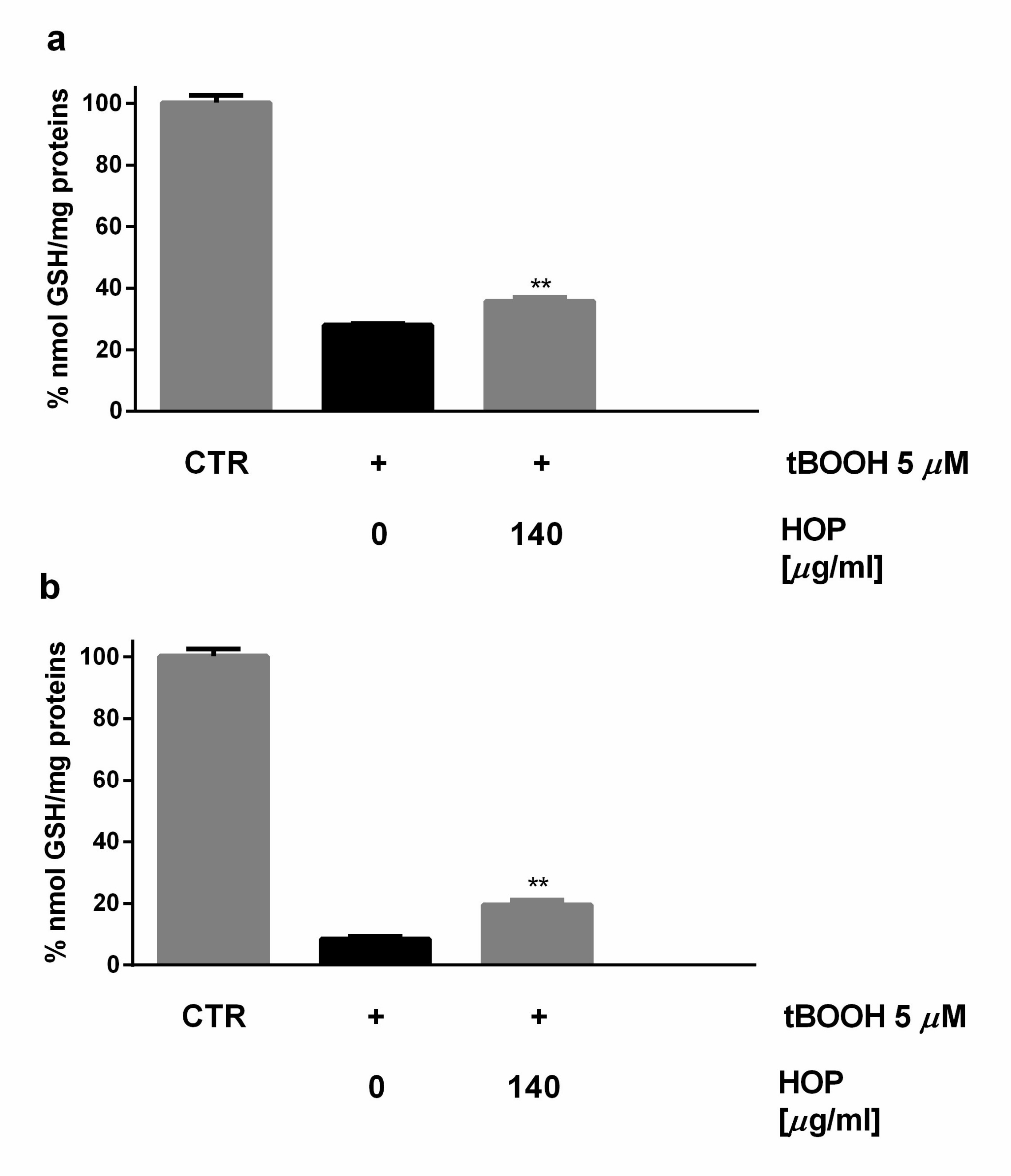

Figure S5: Morphology of A549 cells treated with tBOOH or tBOOH and HOP extract (140 μg/mL) under the co-treatment plus post-treatment protocol.

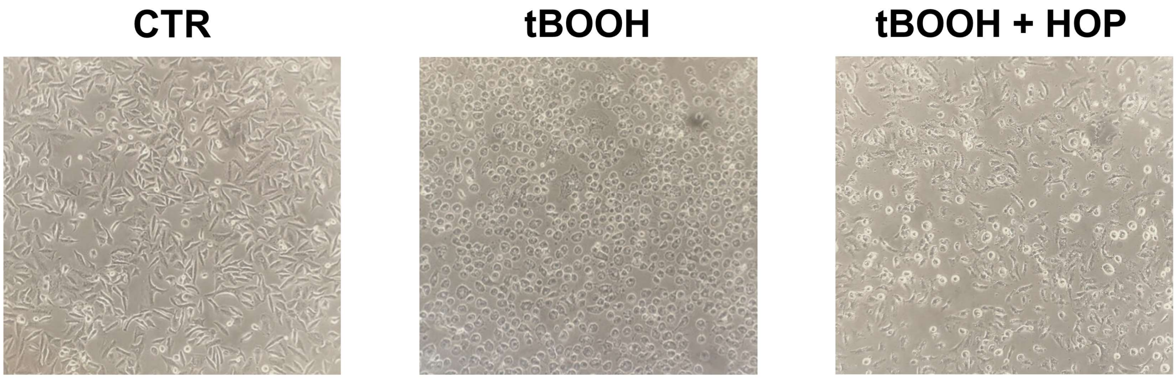

Supplement: Supplementary Materials — Figure S1: high-performance thin-layer chromatography (HPTLC) of the polyphenolic compounds of the HOP extract. (a) Visualization at 366 nm without derivatization. (b) Visualization at 366 nm after natural product reagent (NPR) derivatization. (c) Visualization under white light after NPR and anisaldehyde derivatization. (d) Visualization at 366 nm after anisaldehyde/NPR derivatization. (e) Visualization at 255 nm. Figure S2: effect of the HOP extract on replication of different influenza viral strains in A549 cells. (a) Viral titer measured in supernatants of A549 cells infected with pH1N1, NWS, or ULSTER strain, following incubation of the virus with 140 μg/ml of HOP extract. (b) Western blot analysis of viral proteins from samples obtained as described in (a). Figure S3: effect of the HOP extract (60–140 μg/ml) on the oxidative damage induced by tert-butyl hydroperoxide (tBOOH; 5 μM) in A549 (a) and Caco-2 cells (b). Figure S4: GSH levels measured in A549 cell lysates treated with tBOOH or tBOOH and HOP extract (140 μg/ml) under pretreatment (a) and cotreatment plus posttreatment (b) protocols. Figure S5: morphology of A549 cells treated with tBOOH or tBOOH and HOP extract (140 μg/ml) under the cotreatment plus posttreatment protocol. [file 5919237.f1.pdf]
